# Inhibition of EZH2 exerts antitumorigenic effects in renal cell carcinoma via LATS1

**DOI:** 10.1002/2211-5463.13579

**Published:** 2023-03-21

**Authors:** Seong Hwi Hong, Hyun Ji Hwang, Da Hyeon Son, Eun Song Kim, Sung Yul Park, Young Eun Yoon

**Affiliations:** ^1^ Department of Urology Hanyang University College of Medicine Seoul Korea; ^2^ Department of Translational Medicine Hanyang University Graduate School of Biomedical Science & Engineering Seoul Korea

**Keywords:** EZH2, Hippo pathway, LATS1, renal cell carcinoma, tazemetostat

## Abstract

The most common type of kidney cancer in adults is renal cell carcinoma (RCC), which accounts for approximately 90% of cases. RCC is a variant disease with numerous subtypes; the most common subtype is clear cell RCC (ccRCC, 75%), followed by papillary RCC (pRCC, 10%) and chromophobe RCC (chRCC, 5%). To identify a genetic target for all subtypes, we analyzed The Cancer Genome Atlas (TCGA) databases of ccRCC, pRCC, and chromophobe RCC. Enhancer of zeste homolog 2 (EZH2), which encodes a methyltransferase, was observed to be significantly upregulated in tumors. The EZH2 inhibitor tazemetostat induced anticancer effects in RCC cells. TCGA analysis revealed that large tumor suppressor kinase 1 (LATS1), a key tumor suppressor of the Hippo pathway, was significantly downregulated in tumors; the expression of LATS1 was increased by tazemetostat. Through additional experiments, we confirmed that LATS1 plays a crucial role in EZH2 inhibition and has a negative association with EZH2. Therefore, we suggest that epigenetic control could be a novel therapeutic strategy for three subtypes of RCC.

## Graphical abstract

Overexpressed EZH2 induces methylation of the LATS1 promoter and suppresses the expression of the tumor suppressor LATS1. Reduced LATS1 promotes the transcription of yes‐associated protein 1, a downstream gene, and induces RCC growth. However, the inactivation of EZH2 by the tazemetostat, an EZH2 inhibitor, suppresses RCC growth by blocking the methylation of the LATS1 promoter.

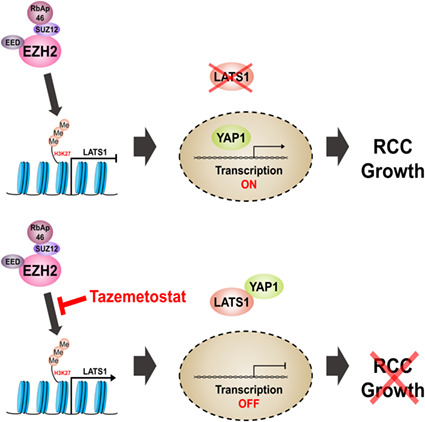

**Abbreviations**
ChIP: chromatin immunoprecipitation
ccRCC: clear cell RCC
EZH2: enhancer of zeste homolog 2
FITC: fluorescein isothiocyanate
H3K27me3: trimethylation of histone 3 lysine 27
IHC: immunohistochemistry
LATS1: large tumor suppressor kinase 1
MSigDB: molecular signatures database
PI: propidium iodide
pRCC: papillary RCC
RCC: renal cell carcinoma
TCGA: The Cancer Genome Atlas
TKIs: tyrosine kinase inhibitors
YAP1: yes‐associated protein 1

Although kidney cancer comprises only 2–3% of all cancers worldwide [1], it is the 10th most frequent cancer among men and women. The most common type of kidney cancer in adults is renal cell carcinoma (RCC), accounting for approximately 90% of cases. RCC is a variant disease with numerous subtypes; the most common subtype is clear cell RCC (ccRCC, 75%), followed by papillary RCC (pRCC, 10%) and chromophobe RCC (chRCC, 5%) [2]. Most kidney cancer research is limited to ccRCC to account for the largest portion of RCC cases. In clinical practice, treatment methods for non‐ccRCC are not standardized and are still considered in clinical trials according to the National Cancer Network guidelines. Therefore, it is important to discover new therapeutic targets that can encompass multiple subtypes of RCC.

Enhancer of zeste homolog 2 (EZH2) is a methyltransferase that catalyzes trimethylation of H3K27 (histone 3 lysine 27) as a major factor of polycomb repressive complex 2 involved in epigenetic gene silencing [3, 4]. It is known to regulate the expression of genes involved in cell differentiation and development and to cause cancer by inhibiting the transcription of tumor suppressor genes [5]. It is overexpressed in various cancers, including breast cancer [6], glioblastoma [7], bladder cancer [8], liver cancer [9], and lung cancer [10, 11]. Currently, tazemetostat, a selective inhibitor of EZH2, is undergoing clinical trials in urinary cancers including bladder cancer (NCT04019327) and prostate cancer (NCT04019327). However, although a recent study reported the potential of EZH2 as a prognostic marker related to the survival rate of RCC patients [12], the effect and underlying mechanism of tazemetostat are unknown in RCC.

The Hippo pathway is not only involved in regulating the size of an animal's organs through cell growth and death but also plays an important role in regulating metabolic homeostasis [13]. LATS1 is a key component of the Hippo pathway [14]. When the Hippo pathway is turned on, LATS1 induces the degradation of YAP/TAZ, thereby maintaining a state in which cancer cell growth and metastasis are inhibited. However, turning off the Hippo pathway inhibits the degradation of YAP/TAZ, which then enters the nucleus and acts as a transcription cofactor that promotes the transcription of genes, especially oncogenes, leading to cancer growth and metastasis [14, 15].

In this study, we discovered key factors that can target multiple subtypes of RCC and ccRCC. We aimed to assess the effects of an EZH2 inhibitor on the Hippo pathway to better understand epigenetic modulators and identify a common drug target for the major RCC subtypes.

## Materials and methods

### Cell culture

The human ccRCC cell line Caki‐1 and the pRCC cell line ACHN were purchased from Korean Cell Line Bank (Seoul, South Korea). The immortalized human chRCC cell line UOK‐276 was kindly provided by W. Marston Linehan (National Cancer Institute, Maryland, USA). All cell lines were maintained in RPMI‐1640 or Dulbecco's modified Eagle medium (Sigma‐Aldrich, St. Louis, Missouri, USA) supplemented with 10% fetal bovine serum (FBS; Sigma‐Aldrich) and 1× antibiotic‐antimycotic (Thermo Fisher, Waltham, Massachusetts, USA). All cells were cultured in an incubator designed to maintain a constant temperature and high humidity for the growth of cells under 5% CO_2_ atmosphere.

### Cell viability

Cell viability was measured using EZ‐CYTOX (DoGenBio, #EZ‐1000, Seoul, Korea). RCC cells were grown to 50% confluency in 96‐well plates (SPL, Kyonggi‐do, South Korea) and treated with 0 to 100 μm tazemetostat for 24 h at 37 °C. Following incubation, 10 μL of EZ‐CYTOX solution was added to each well. Absorbance at a wavelength of 450 nm was determined by a microplate reader (BIO‐RAD, #1681130, Hercules, California, USA). The cell viability rates were calculated, and graphs were generated. The cell viability was evaluated based on the treated group versus the untreated group results.

### RNA extraction and reverse transcription PCR

Using TRIzol reagent, total RNA was extracted and reverse transcribed using an AccuPower RT PreMix kit (Bioneer Daejeon, South Korea). For qRT‐PCR analysis, cDNAs were amplified with LightCycler® 480 SYBR Green I Master Mix (Roche, Basel, Switzerland). Using a LightCycler® 480 II PCR system (Roche), the cycle threshold value (*C*t value) and the exponential amplification time of the PCR product were monitored in real time. cDNAs were amplified using the following primers: RT_*GAPDH*_forward, 5′‐GAG TCA ACG GAT TTG GTC GT; RT_*GAPDH*_reverse, 3′‐TGG AAG ATG GTG ATG GGA TT; RT_*LATS1*_forward, 5′‐CTC CAC CAC CTC TCA ACA CT; and RT_*LATS1*_reverse, 3′‐CGA TTC ACA GTG CCA GCA G. PCR conditions consisted of a predenaturation step of 95 °C for 5 min, followed by 60 cycles of 95 °C for 1 min, 60 °C for 1 min, and 72 °C for 1 min, with a final extension of 72 °C for 10 min.

### Apoptosis assay

The apoptosis rate was assessed using the annexin V–fluorescein isothiocyanate (FITC) apoptosis detection kit (BD Biosciences, #556547, Franklin Lakes, New Jersey, USA). Cells were transfected with siLATS1 and treated with various concentrations of tazemetostat for a further period of 24 h. For each sample, the collected supernatant and trypsinized cells were rinsed in Dulbecco's phosphate‐buffered saline and suspended in 1× binding buffer at a concentration of 1 × 10^6^ cells/mL. Then, 5 μL FITC‐conjugated annexin V and 2 μL propidium iodide (PI) were added to 100 μL containing 1 × 10^5^ cells and incubated for 15 min at room temperature in the dark. After incubation, 400 μL binding buffer (1×) was added to each tube, and the cells were analyzed using a FACSCanto flow cytometer (BD Biosciences).

### Western blot analysis

Cells were lysed with protein lysis radioimmunoprecipitation assay buffer and briefly sonicated for complete lysis of protein. Cell lysates were boiled with 4× sample buffer, and 30 μg of each protein was separated by sodium dodecyl sulfate–polyacrylamide gel electrophoresis. For the quantitation of protein, the lysate was measured by bicinchoninic acid assay. Antibodies against EZH2 (1 : 1000, #195409, Abcam, Cambridge, UK), LATS1 (1 : 1000, Cell Signaling Technology, #3477S, Danvers, Massachusetts, USA), yes‐associated protein 1 (YAP1; 1 : 1000, Cell Signaling Technology, #14074S), H3K27me3 (1 : 500, Abcam, #195477), histone H3 (1 : 1000, Abcam, #ab1791), beta‐actin (1 : 1000, GeneTex, #GTX109639, Irvine, California, USA) were purchased from the indicated companies. We analyzed the protein expression using ChemiDoc XRS+ with image lab software and quantitated the band using imagej software (version 1.53t) [16].

### Chromatin immunoprecipitation assay

For the chromatin immunoprecipitation (ChIP) assay, we used Pierce™ Agarose ChIP kit (Thermo Fisher, #26156). ChIP assays were performed according to instructions from the manufacturer. Briefly, 50 μg DNA fragmented to 200–500 bp in size by enzymatic digestion was precleared with protein A magnetic beads. The precleaned DNA was immunoprecipitated with EZH2 (1 μL, Abcam, #195409) and H3K27me3 (1 μL, Abcam, #195477). After this, DNA extraction was performed for chromatin fragments, and qPCR was performed on the DNA bound to each target protein using LightCycler 480 II (Roche). Immunoglobin G experiments as the negative control were conducted for all samples. The qPCR results were calculated as IP/1% input as follows: (IP − IgG)/(Input − IgG). The following specific primers were used for ChIP‐qPCR of chromatin fragments: ChIP_LATS1 promoter_forward, CGC TCA CGA ACG ATC AGA; ChIP_LATS1 promoter_reverse, GAC CTG GCT CTC CCC TTA AC.

### Wound healing assay

Cells were seeded to an even cell density and grown to 80% confluency in 6‐well cell culture plates. After overnight incubation in a serum‐free medium, cell layers were scratched with sterile yellow tips. The cell images for the width of the initial gap (0 h) and the residual gap at 16 or 24 h after wounding were captured and measured using an optical microscope at 40× magnification (Nikon, Eclipse TS100, Tokyo, Japan).

### Invasion assay

For the invasion assay, cell motility was measured using a Boyden chamber‐like design (BD Biosciences). To coat the upper surface of the transwell chamber, 100 μL Matrigel (0.3 mg·mL^−1^, BD Biosciences) was used. After coating for 3 h at 37 °C, the remaining supernatant was removed, 1 × 10^6^/500 μL cell suspension was placed in the top chamber, and the chambers were placed in a 24‐well plate. As a chemoattractant, 700 μL 20% FBS medium was incubated under the chamber overnight. The next day, the membrane of the chamber was stained using 0.4% crystal violet. The images of the invaded cells were captured using a microscope at 40× magnification (Nikon, Eclipse TS100), and the cell numbers were quantitated in image fields.

### TCGA data analysis

To investigate *EZH2* and *LATS1* levels in a larger RCC cohort, we analyzed RNA‐seq‐based gene expression profiling data from The Cancer Genome Atlas (TCGA) RCC project (KIRC‐ccRCC, KIRP‐pRCC, and KICH‐chRCC) [17, 18, 19]. A log2 transformation for raw data of RNA‐seq was identically applied. Then, the fold change for expression of the tumor and the normal tissues (healthy kidney tissue) was compared. The Molecular Signatures Database (MSigDB) is one of the most widely used and comprehensive databases of gene sets for performing gene set enrichment analysis. Anyone is possible to direct analysis on the website (https://www.gsea‐msigdb.org), and more detailed information for MSigDB can be found in this article [20].

### Immunohistochemistry

Formalin‐fixed paraffin‐embedded tissue samples were prepared using one T1 stage and two T3 stage tumor tissues obtained from three randomly selected patients. The paraffin‐embedded tissues of RCC patients were first cut into <3‐mm‐thick sections to prepare the tissue slides. The slide of each section was deparaffinized with xylene and hydrated by alcohol. To protect disruption after 3,3′‐diaminobenzidine development by endogenous peroxidase activity, the slide was washed in 0.6% H_2_O_2_ in methanol for 15 min. Then, the slides were treated with 0.1% Triton‐X 100, washed three times with phosphate‐buffered saline, and blocked with 10% goat serum. To examine the expressions of EZH2 (1 : 500, Abcam, #195409) and LATS1 (1 : 500, Cell Signaling Technology, #3477S), the slides were treated with a corresponding primary antibody overnight at 4 °C, and the next day, it was incubated with secondary antibody at room temperature. The slide was chromogenic reacted using the 3,3′‐diaminobenzidine kit (Vector Laboratories, Burlingame, California, USA) and counterstained by hematoxylin and bluing solution. A coverslip was mounted on the slide and observed under an optical microscope (Leica DM4000B, Leica Microsystems, Wetzlar, Germany) at 200× magnification. Using software leica application suite version 4.1.0, the stained area relative to the area of total tissue was calculated and quantified as a percentage.

### Transient transfections

The next day after seeding RCC cells in 6‐well plates, they were transiently transfected using 100 nm siRNA (Bioneer, #9113, Daejeon, South Korea) and Lipofectamine 2000 reagent (Thermo Fisher). After overnight incubation, Opti‐MEM was replaced with an antibiotic‐free growth medium.

### Statistical analyses

All experiments were performed in triplicate, and all data were analyzed in triplicate. The results are indicated as the means ± standard deviation. Statistical calculations and comparisons were completed using a *t*‐test or one‐way analysis of variance and Tukey's *post hoc* test with graphpad prism 8.0 (GraphPad, San Diego, California, USA). A *P*‐value <0.05 was considered statistically significant.

### Ethics

Institutional Review Board Statement: The study was conducted according to the guidelines of the Declaration of Helsinki and approved by the Institutional Review Board of Hanyang University Hospital (protocol code number #HYUH 2019‐12‐002‐007, 06‐DEC‐2021). Written informed consent was obtained from all subjects involved in the study.

## Results

### Epigenetic enzyme *EZH2* is overexpressed in three RCC subtype cohorts and EZH2 inhibition by small‐molecule tazemetostat induces antitumorigenic effects in RCC cells

**Fig. 1:**
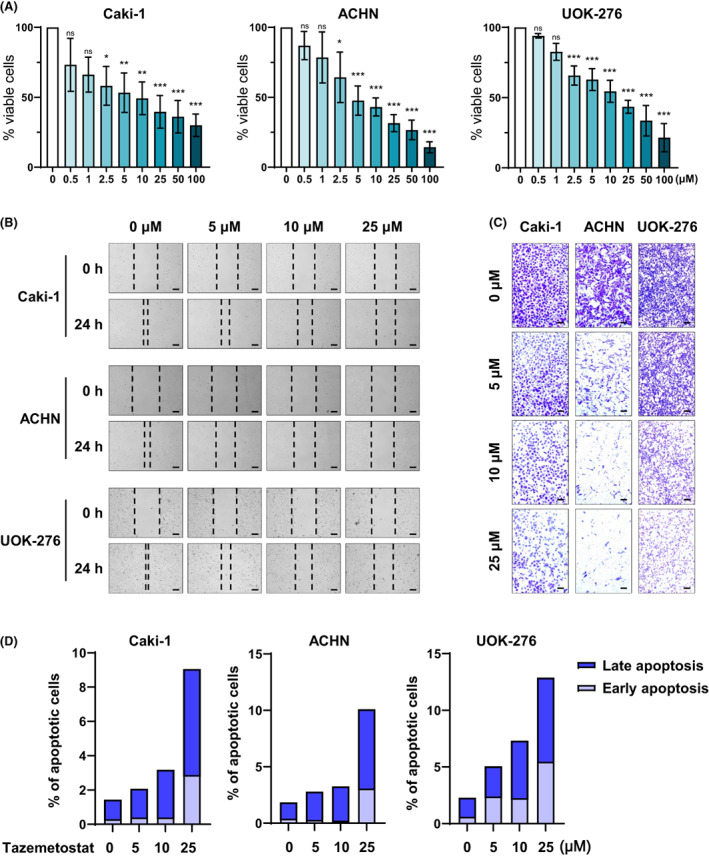
Overexpressed *EZH2* inhibition induces antitumorigenic effect in RCC. (A) Cell viability rate in Caki‐1, ACHN, and UOK‐276 cells with or without tazemetostat treatment (mean ± SD, statistical calculations and comparisons were completed using a one‐way analysis of variance and Tukey's *post hoc* test. *N* = 3; *P < 0.05, **P < 0.01, ***P < 0.001). (B) Wound healing capacity determined for 24 h after tazemetostat treatment in Caki‐1, ACHN, and UOK‐276 cells (scale bars = 250 μm). (C) Representative images of invaded cells that passed through transwell chambers in invasion assays using matrigel (0.4% crystal violet staining, 40× magnification; scale bars = 250 μm). (D) Percentage of apoptotic cells based on annexin V–FITC/PI staining assay, and representative images for fluorescence‐activated single cell sorting analysis performed after tazemetostat treatment in Caki‐1, ACHN, and UOK‐276 cells.

The Cancer Genome Atlas database was analyzed to find targets for ccRCC, pRCC, and chRCC. Among the genes with more than 1.5‐fold change in common in the three RCCs, 1850 genes were downregulated and 156 genes were upregulated in tumor tissue compared with nontumor tissue. In RCCs, 156 upregulated genes were classified into eight categories according to their role using gene family categorization provided by the MSigDB in gene set enrichment analysis (https://www.gsea‐msigdb.org) (Fig. S1A). Among the eight categories, we tried to find a main target of RCCs in transcription factors because it can directly affect the transcription of other genes. Recently, because interest in epigenetic regulators for cancer treatment is increasing, EZH2 as the only epigenetic enzyme was selected in 23 transcription factors that satisfy all conditions. We observed that *EZH2* was significantly increased in tumors compared with nontumor (Fig. S1B). Because tazemetostat is a potent inhibitor of EZH2 [21], its sensitivities to ccRCC (Caki‐1), pRCC (ACHN), and chRCC (UOK‐276) cell lines were assessed. To investigate the role of EZH2 in cell proliferation, we measured cell viability using a colorimetric assay, which is an MTT‐based assay. Tazemetostat dose‐dependently reduced the growth rate of all three cell lines (Fig. 1A). Consistent with this result, in scratch wound healing and invasion experiments, tazemetostat diminished wound healing capability and invasion in Caki‐1, ACHN, and UOK‐276 cells (0, 5, 10, 25 μm: 1.9, 35.4, 43.7, and 78.5% for Caki‐1, 16.9, 83.7, 91.7, and 94.9% for ACHN, and 1.8, 47.2, 57.6, and 73.6% for UOK‐276) (Fig. 1B,C, Fig. S2A). To further study the antitumorigenic effect of tazemetostat, we also examined apoptosis and found increased apoptosis effects in RCC cell lines (0, 5, 10, 25 μm: 1.4, 2.1, 3.2, and 9.1% for Caki‐1; 1.8, 2.8, 3.3, and 10.1% for ACHN; and 2.3, 5.1, 7.3, and 12.9% for UOK‐276, respectively) (Fig. 1D and Fig. S2B). The cumulative results reveal that EZH2 could be a remedial strategic target in three major types of RCC and that the inactivation of EZH2 by tazemetostat has anticancer effects in RCC cell lines.

### LATS1 of the Hippo pathway is the main target of EZH2 inhibition in RCCs

**Fig. 2:**
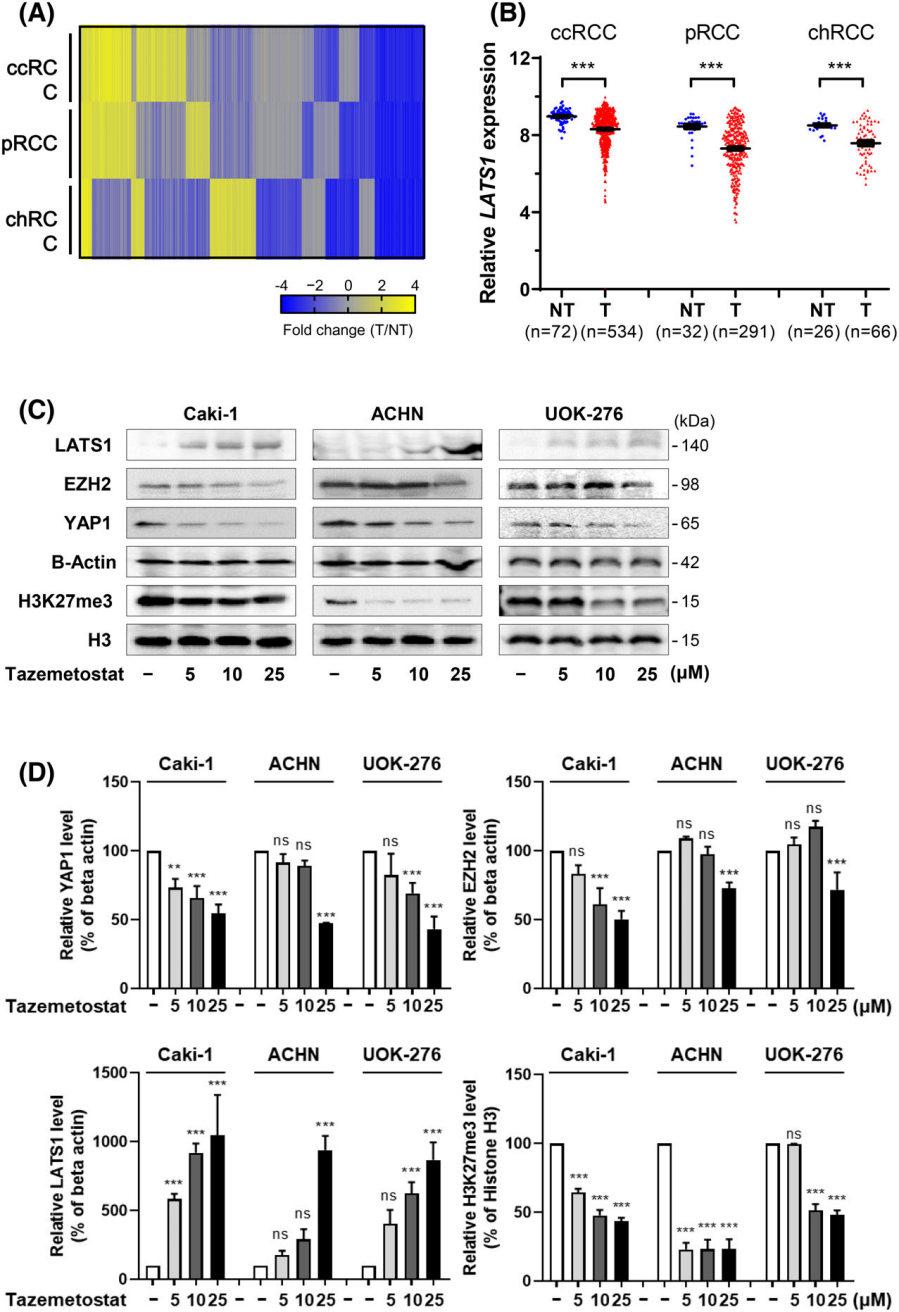
LATS1 of the Hippo pathway is the main target of EZH2 inhibition in RCC. (A) Significant downregulation of genes (*n* = 1850) in tumor compared with nontumor tissues in three types of RCC from the TCGA database. (B) Significant downregulation of *LATS1* expression in tissues of patients with RCC (ccRCC, pRCC, and chromophobe; chRCC) from the TCGA database (mean ± SD, statistical calculations and comparisons were completed using a *t*‐test) [***P < 0.001, tumor (T) versus nontumor (NT) tissue]. (C, D) The protein levels of LATS1, EZH2, YAP1, and H3K27me3 detected by western blot in RCC cell lines. As a loading control, beta‐actin and histone H3 were used (mean ± SD, statistical calculations and comparisons were completed using a one‐way analysis of variance and Tukey's *post hoc* test. *N* = 3) (**P < 0.01, ***P < 0.001).

Since the main role of overexpressed EZH2 in cancer is to suppress the expression of tumor suppressor genes [22, 23], we predicted that the expression of the tumor suppressor gene can be recovered by EZH2 inactivation in RCCs. To find out what tumor suppressors are increased by tazemetostat, we focused on genes that were downregulated in tumor compared with nontumor tissues through TCGA analysis (Fig. 2A). We observed that the large tumor suppressor kinase 1 (*LATS1*), a key tumor suppressive gene of the hippo pathway, was commonly downregulated in the three subtypes of RCC (mean value of NT and T: 8.97 and 8.31 for ccRCC; 8.45 and 7.31 for pRCC, and 8.51 and 7.58 for chRCC) (Fig. 2B). Tazemetostat treatment induced a decrease in global levels of trimethylation of histone 3 lysine 27 (H3K27me3) (0, 5, 10, 25 μm for H3K27me3: 100, 64.7, 47.9, and 43.6% for Caki‐1, 100, 23.2, 23.4, and 23.6% for ACHN, and 100, 99.6, 51.6, and 48.1% for UOK‐276), an increase in LATS1 expression (0, 5, 10, 25 μm for LATS1: 100, 584.9, 920.2, and 1064% for Caki‐1, 100, 177.9, 292.8 and 940.6% for ACHN, and 100, 405.4, 627.1, and 868.6% for UOK‐276), and a decrease in YAP1 expression (0, 5, 10, 25 μm for YAP1: 100, 73.3, 66, and 54.8% for Caki‐1, 100, 91.6, 89.2, and 47.5% for ACHN, and 100, 82.6, 69.4, and 43.3% for UOK‐276), known as a negative downstream target of LATS1 in the hippo pathway, in a dose‐dependent manner (Fig. 2C,D, and Fig. S2C).

### Loss of LATS1 interferes with anticancer effects by EZH2 inactivation

**Fig. 3:**
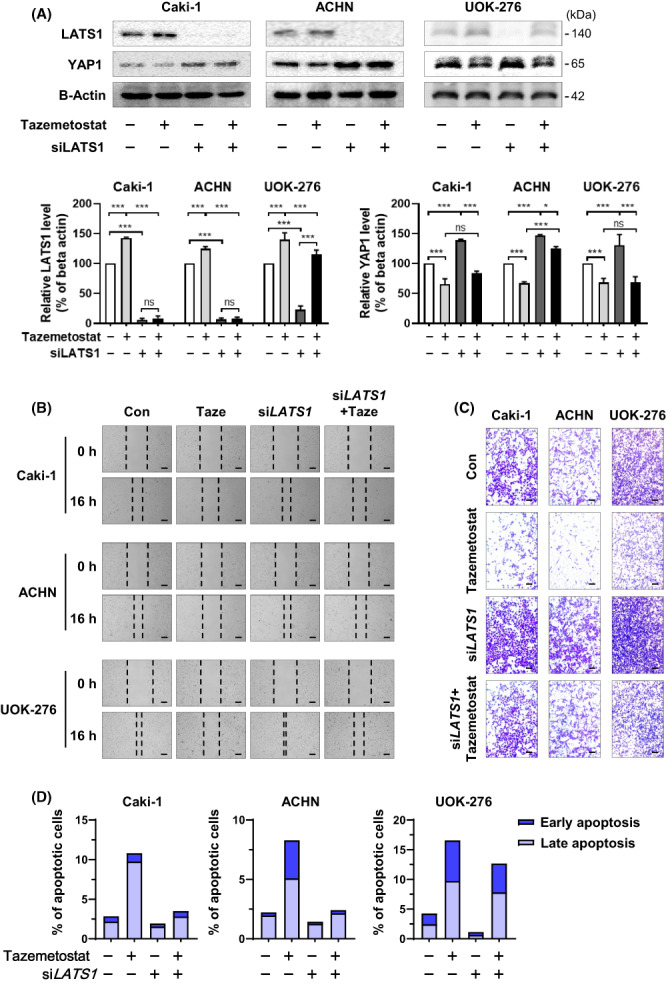
Loss of LATS1 interferes with anticancer effects by EZH2 inactivation. (A) Endogenous protein expression levels of LATS1 and YAP1 were determined by western blot in RCC cell lines. As a loading control, beta‐actin was used to quantify western blot data (mean ± SD, statistical calculations and comparisons were completed using a one‐way analysis of variance and Tukey's *post hoc* test. *N* = 3) (*P < 0.05, ***P < 0.001). (B) Wound healing capacity determined for 16 h after treatment of 25 μm tazemetostat and loss of LATS1 in Caki‐1, ACHN, and UOK‐276 cells (scale bars = 250 μm). (C) Representative images of invaded cells that passed through transwell chambers in invasion assay using matrigel (0.4% crystal violet staining, 40× magnification) (scale bars = 250 μm). (D) Annexin V–FITC/PI staining assay and the representative images for fluorescence‐activated single cell sorting analysis performed after 25 μm tazemetostat treatment and loss of *LATS1* in Caki‐1, ACHN, and UOK‐276 cells.

To further investigate whether EZH2 is essential for the suppression of LATS1, we performed a loss‐of‐function study by knockdown of LATS1 cells using siRNA in Caki‐1, ACHN, and UOK‐276 RCC cells (Fig. S3A). As shown in Fig. 3A and Fig. S3B, increased LATS1 expression was evident after tazemetostat treatment (control, tazemetostat, si*LATS1*, tazemetostat + si*LATS1*: 100, 142.9, 6.1, and 8.5% for Caki‐1; 100, 124.8, 6.8 and 8.5% for ACHN; and 100, 139.9, 23 and 114.9% for UOK‐276, respectively). Furthermore, LATS1 loss recovered the antitumorigenic effect of tazemetostat including reduced wound healing capability and invasion ability (control, tazemetostat, si*LATS1*, tazemetostat + si*LATS1*: 43.4, 74.1, 29.7, and 57.1% for Caki‐1; 33.1, 84, 19.9 and 36.3% for ACHN; and 18.9, 71.9, 4.7 and 38.8% for UOK‐276, respectively) (Fig. 3B,C and Fig. S3C). Increased RCC apoptosis by tazemetostat was rescued by inhibition of *LATS1* (control, tazemetostat, si*LATS1*, tazemetostat + si*LATS1*: 2.8, 10.8, 1.9, and 3.5% for Caki‐1; 2.2, 8.3, 1.4, and 2.4% for ACHN; and 4.3, 16.6, 1.1, and 12.7% for UOK‐276, respectively) (Fig. 3D and Fig. S3D). Overall, the results indicate that LATS1 is a direct target of the epigenetic regulator EZH2 in three types of RCC cells.

### Tazemetostat downregulates LATS1 directly via suppression of EZH2 binding at the LATS1 promoter

**Fig. 4:**
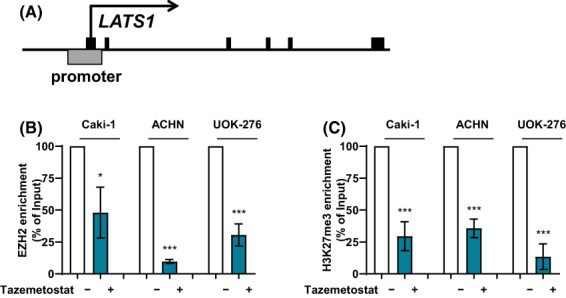
Tazemetostat downregulates *LATS1* directly via suppression of EZH2 binding at the *LATS1* promoter. (A) Schematic diagram of the *LATS1* promoter. On the line, the black boxes indicate exon, and the gray box indicates the *LATS1* promoter region for a specific primer. Chromatin digested with enzymes was immunoprecipitated using anti‐EZH2 and anti‐H3K27me3 antibodies. (B, C) In the *LATS1* regulatory region, changes for the (B) binding of EZH2 and (C) enrichment of H3K27me3 by 25 μm tazemetostat treatment based on qPCR analysis (mean ± SD, statistical calculations and comparisons were completed using a one‐way analysis of variance and Tukey's *post hoc* test. *N* = 3) (*P < 0.05, ***P < 0.001).

To examine whether the upregulation of LATS1 by tazemetostat is a result of direct regulation by inhibiting catalyzation for H3K27me3 on the *LATS1* promoter, we performed ChIP using EZH2 and H3K27me3 antibodies (Fig. 4A). EZH2 was highly enriched at the promoter of *LATS1* in three cell lines, and it decreased upon treatment (24 h) with tazemetostat (tazemetostat treatment: 48.0 in Caki‐1; 9.7% in ACHN; and 30.5% in UOK‐276, respectively) (Fig. 4B). To delineate the epigenetic role of the *LATS1* promoter after tazemetostat treatment, ChIP was performed using H3K27me3 antibody, a well‐known suppressive promoter marker. *LATS1* promoter regions were hypomethylated by EZH2 inactivation (tazemetostat treatment: 29.5 in Caki‐1; 35.6% in ACHN; and 13.5% in UOK‐276, respectively) (Fig. 4C), which corresponded with *LATS1* expression state (Fig. 2C). These results indicate that the epigenetic component EZH2 was correlated with *LATS1* transcription and *LATS1* is a direct target of EZH2 in RCC cells.

### High EZH2 and low LATS1 expression are associated with poor outcomes in RCC patients

**Fig. 5:**
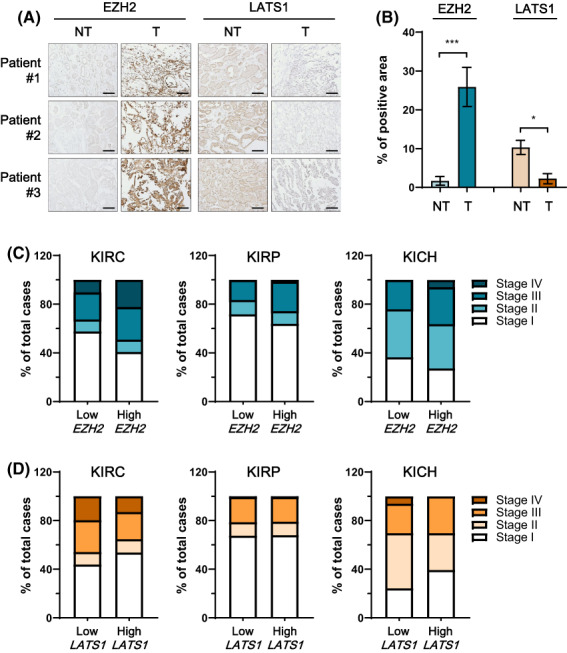
High *EZH2* and low *LATS1* expression are associated with poor outcomes in RCC patients. (A) Representative image showing immunohistochemical analysis for EZH2 and H3K27me3 antibodies in healthy and tumor tissues from three patients (scale bars = 100 μm). (B) Percentage of positive area in tumor and nontumor tissues immunohistochemically stained with anti‐EZH2 and anti‐H3K27me3 antibodies analyzed using las version 4.1.0 software (mean ± SD, statistical calculations and comparisons were completed using a one‐way analysis of variance and Tukey's *post hoc* test. *N* = 3) (*P < 0.05, ***P < 0.001). (C, D) Distribution of the clinical stages relative to (C) *EZH2* and (D) *LATS1* mRNA expression in RCCs.

To verify the association between EZH2 and LATS1 expression, immunohistochemistry (IHC) was performed using tissue from RCC patients. These IHC data showed that EZH2 is more highly expressed and LATS1 is more lowly expressed in human RCC tissues than healthy tissues (NT/T: 1.7/25.9 for EZH2 and 10.3/2.2 for LATS1) (Fig. 5A,B). Furthermore, to verify the association between EZH2 and LATS1 expression with public big data, we analyzed clinicopathological parameters based on TCGA cohort (mRNA) and TNM stage for ccRCC, pRCC, and chRCC. Among all samples from the TCGA cohort, some cases were missing part of the clinicopathological data. The median value for mRNA expression of *EZH2* and *LATS1* among all samples was chosen as a cutoff to classify the samples into high‐ and low‐expression groups. We noticed that in the TCGA cohort, tumors of the high *EZH2* expression group were positively associated with stage IV in ccRCC (stage I, 40.78%; stage IV; 22.35%), pRCC (stage I, 63.89%; stage IV; 1.39%), and chRCC (stage I, 27.27%; stage IV; 6.06%) (Fig. 5C). However, the percentage of the high *LATS1* expression group was negatively associated with stage IV in ccRCC (stage I, 53.73%; stage IV; 12.94%), pRCC (stage I, 68.06%; stage IV; 0.69%), and chRCC (stage I, 39.39%; stage IV; 0%) (Fig. 5D). Although the ratios of stages according to the expression of *LATS1* in pRCC do not appear to be different on the graph, the ratios between the high *LATS1* expression group (stage I/II; 79.17%, stage III/IV; 20.83%) and the low *LATS1* expression group (stage I/II; 78.62%, stage III/IV; 21.38%) are slightly different.

## Discussion

**Fig. 6:**
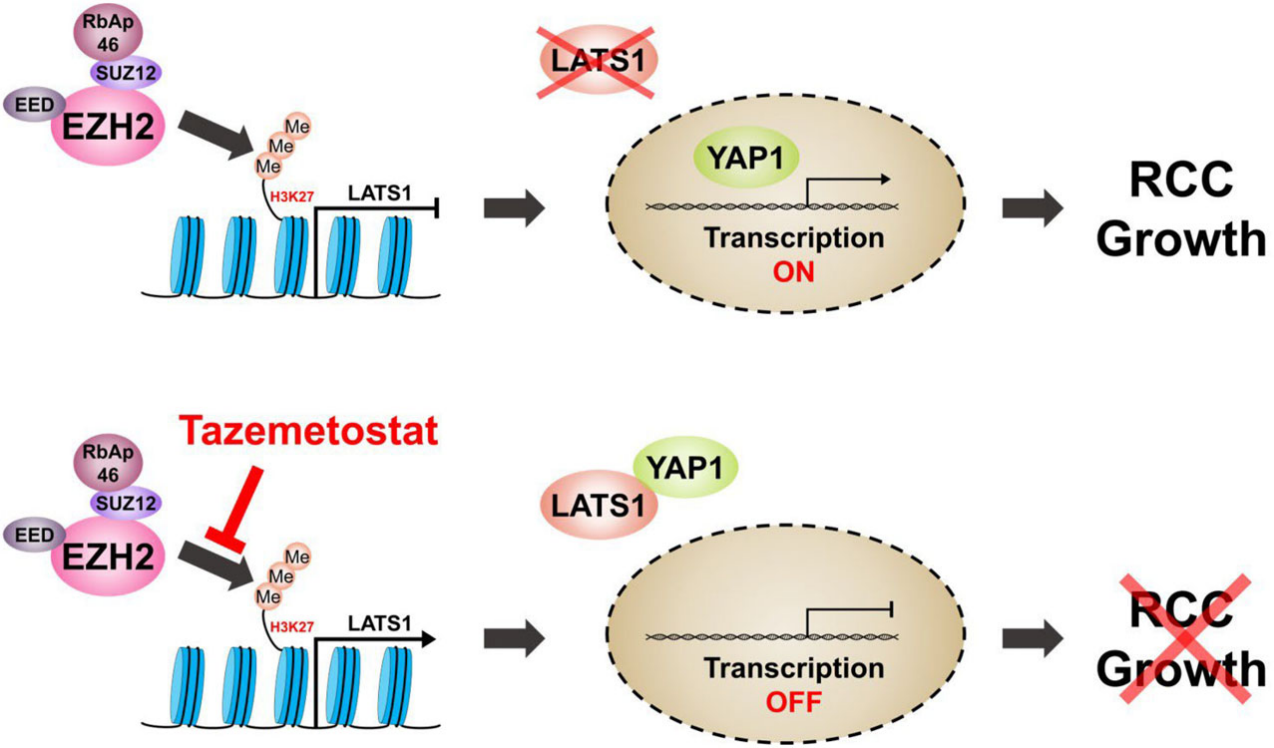
Overexpressed EZH2 suppresses expression of LATS1 by inducing methylation in LATS1 promoter. Decreased LATS1 promotes the transcription of YAP1, a downstream gene, and induces RCC growth. However, the inactivation of EZH2 by tazemetostat inhibits RCC growth by blocking the methylation of LATS1 promoter.

We investigated the activity of an EZH2 inhibitor in RCC. Our findings revealed LATS1 and Hippo pathway regulation as major targets of EZH2 inhibition (Fig. 6). This study found that EZH2 is overexpressed in RCC tissues compared with healthy tissues in three large cohorts, as well as in RCC cell lines. EZH2 inhibition by tazemetostat induced antitumorigenic effects including increased apoptosis, reduced wound healing capacity, and inhibition of invasive ability in RCC cell lines. The results of ChIP assays with antibodies against EZH2 and H3K27me3 and loss‐of‐function experiments against LATS1 indicate that EZH2 directly modulates the transcription of LATS1. In contrast to EZH2, LATS1 expression was downregulated in three large cohorts of RCC patients, suggesting that LATS1 is a direct target of EZH2. Lastly, EZH2 expression was significantly associated with a higher stage of RCC, indicating that the EZH2‐LATS1 axis is an important target in patients with RCCs. Taken together, this study demonstrated that targeting epigenetic enzymes with EZH2 inhibitors will be an innovative therapeutic target for LATS1‐underexpressed patients with RCC.

As the name suggests, ccRCC is characterized by cells with histologically empty cytoplasm because lipids and glycogen accumulated in the cell are removed during the histological process [24]. The study by Tun et al. [25] revealed that adipogenic genes change when renal epithelial cells are converted into tumors. Although lipid reprogramming in ccRCC is a well‐known phenomenon, the importance of this process is still unclear. It is known that the Hippo pathway not only regulates the oncogene by translocation of YAP1 to the nucleus but also has complex regulatory functions related to the metabolism of various types of energy such as glucose [26, 27, 28, 29] and lipids [30]. Although this study did not assess downstream genes of the Hippo pathway, this would be an important avenue for future study to determine how the EZH2‐regulated Hippo pathway regulates metabolism given its crucial role in energy metabolism in RCC. In addition, ccRCC is sensitive to changes in glucose or lipid metabolism, whereas pRCC or chRCC can be controlled by other pathways, so further study is necessary. Because we used the TCGA database for the three types of RCC in our gene candidate discovery process, our study is not limited to ccRCC, which is a limitation of existing RCC treatment. Therefore, our results may provide the basis for studying how glucose or lipid metabolism is modulated in other RCCs.

In addition, tyrosine kinase inhibitors (TKIs), drugs widely used for the treatment of RCC, mainly inhibit angiogenesis [31], and complete tumor remission and sufficient survival gain are difficult to achieve with these drugs. In the case of sunitinib, a representative TKI, resistance has been noted as a problem for the last decade [32, 33, 34]. A recent study revealed that the mechanism for the acquisition of sunitinib resistance is closely associated with EZH2 [35]. Based on this research, the combination therapy of tazemetostat and the drugs currently used for RCC could be a new treatment strategy for RCC.

In several studies, the advantage of EZH2 as a target in cancer was reported, and especially, Nitya et al. reported that tazemetostat had the lowest *in vitro* IC50 as well as Methyl IC50 compared with GSK126 and CPI‐1205 among EZH2 inhibitors currently in preclinical or early clinical trials [36, 37]. Research is being conducted to show that tazemetostat is useful for combination therapy with many drugs in various cancers, and tazemetostat was suggested as a potential drug to be used in combination with immunotherapy such as pembrolizumab (NCT03854474). So, it is necessary to optimize the combination of tazemetostat with various drugs including TKI or immunotherapy. However, there is research that tazemetostat is an effective compound but not suitable for patients with renal injury [38]. So it is essential to check the renal injury and review the drug response before applying combination therapy or treatment using tazemetostat.

Taken together, we revealed that *EZH2* as an epigenetic enzyme is overexpressed in three types of RCCs. To our knowledge, this study is the first to demonstrate that an EZH2 inhibitor suppresses the inhibition of LATS1 transcription and its downstream pathway in RCCs. In further studies, it would be intriguing to examine the intricate connection between tazemetostat and other drugs, which would undoubtedly shed light on the physiological significance for the treatment of various cancers due to the dysregulation of epigenetic components. Also, we expect these results to bring significant benefits for time and cost to the field and treatment for RCC patients.

## Author contributions

SHH and YEY involved in research conception and design. SHH and HJH involved in data acquisition. SHH and HJH involved in statistical analysis. SHH and YEY involved in data analysis and interpretation. SHH, SYP, and YEY involved in drafting of the manuscript. SHH and YEY involved in critical revision of the manuscript. HJH, DHS, and ESK involved in administrative, technical, or material support.

## Conflict of interest

The authors declare no conflict of interest.

## Supporting information


**Fig. S1.** *EZH2* is overexpressed in renal cell carcinoma (RCC). (A) Analysis of gene family categorized to provide a functional overview for 156 genes with increased expression in tumor compared with nontumor tissues. (B) Significant overexpression of *EZH2* in the RCC tissues of three cohorts (ccRCC, pRCC, and chRCC) from the TCGA database (mean ± SD, statistical calculations and comparisons were completed using a *t*‐test) (**P < 0.01 and ***P < 0.001, versus nontumor tissue).
**Fig. S2.** Overexpressed EZH2 inhibition induces growth suppression in RCCs. (A) Annexin V–fluorescein isothiocyanate (FITC)/propidium iodide (PI) staining assay and the representative images for fluorescence‐activated single cell sorting (FACS) analysis performed after tazemetostat treatment in Caki‐1, ACHN, and UOK‐276 cells. (B) The protein levels of LATS1, EZH2, YAP1, and H3K27me3 detected by western blot in HK‐2 cell lines. As a loading control, beta‐actin and histone H3 were used (mean ± SD, statistical calculations and comparisons were completed using a one‐way analysis of variance and Tukey's *post hoc* test. *N* = 3) (*P < 0.05, **P < 0.01 and ***P < 0.001).
**Fig. S3.** Loss of *LATS1* interferes with apoptosis by EZH2 inactivation. (A) *LATS1* mRNA levels determined by qRT‐PCR after the loss of *LATS1* expression in Caki‐1 cells. (B) Endogenous protein expression levels of LATS1 and YAP1 were determined by western blot in HK‐2 cell lines. As a loading control, beta‐actin was used to quantify western blot data. (C) Representative images for fluorescence‐activated single cell sorting (FACS) analysis. Annexin V–fluorescein isothiocyanate (FITC)/propidium iodide (PI) staining assay was performed after tazemetostat treatment and loss of *LATS1* in Caki‐1, ACHN, and UOK‐276 cells (mean ± SD, statistical calculations and comparisons were completed using a one‐way analysis of variance and Tukey's *post hoc* test. *N* = 3) (*P < 0.05, **P < 0.01 and ***P < 0.001).

## Data Availability

The data presented in this study are available upon request from the corresponding author.
