# Supplementary material for: Inhibition of EZH2 exerts antitumorigenic effects in renal cell carcinoma via LATS1
Source: FEBS Open Bio. 2023 Mar 21;13(4):724–35. doi: 10.1002/2211-5463.13579 (PMC10068324; doi:10.1002/2211-5463.13579)
Supplement: Supplementary file 1 — Fig. S1. EZH2 is overexpressed in renal cell carcinoma (RCC). (A) Analysis of gene family categorized to provide a functional overview for 156 genes with increased expression in tumor compared with nontumor tissues. (B) Significant overexpression of EZH2 in the RCC tissues of three cohorts (ccRCC, pRCC, and chRCC) from the TCGA database (mean ± SD, statistical calculations and comparisons were completed using a t‐test) (**P < 0.01 and ***P < 0.001, versus nontumor tissue). Fig. S2. Overexpressed EZH2 inhibition induces growth suppression in RCCs. (A) Annexin V–fluorescein isothiocyanate (FITC)/propidium iodide (PI) staining assay and the representative images for fluorescence‐activated single cell sorting (FACS) analysis performed after tazemetostat treatment in Caki‐1, ACHN, and UOK‐276 cells. (B) The protein levels of LATS1, EZH2, YAP1, and H3K27me3 detected by western blot in HK‐2 cell lines. As a loading control, beta‐actin and histone H3 were used (mean ± SD, statistical calculations and comparisons were completed using a one‐way analysis of variance and Tukey's post hoc test. N = 3) (*P < 0.05, **P < 0.01 and ***P < 0.001). Fig. S3. Loss of LATS1 interferes with apoptosis by EZH2 inactivation. (A) LATS1 mRNA levels determined by qRT‐PCR after the loss of LATS1 expression in Caki‐1 cells. (B) Endogenous protein expression levels of LATS1 and YAP1 were determined by western blot in HK‐2 cell lines. As a loading control, beta‐actin was used to quantify western blot data. (C) Representative images for fluorescence‐activated single cell sorting (FACS) analysis. Annexin V–fluorescein isothiocyanate (FITC)/propidium iodide (PI) staining assay was performed after tazemetostat treatment and loss of LATS1 in Caki‐1, ACHN, and UOK‐276 cells (mean ± SD, statistical calculations and comparisons were completed using a one‐way analysis of variance and Tukey's post hoc test. N = 3) (*P < 0.05, **P < 0.01 and ***P < 0.001). [file FEB4-13-724-s001.pptx]

## Slide 1
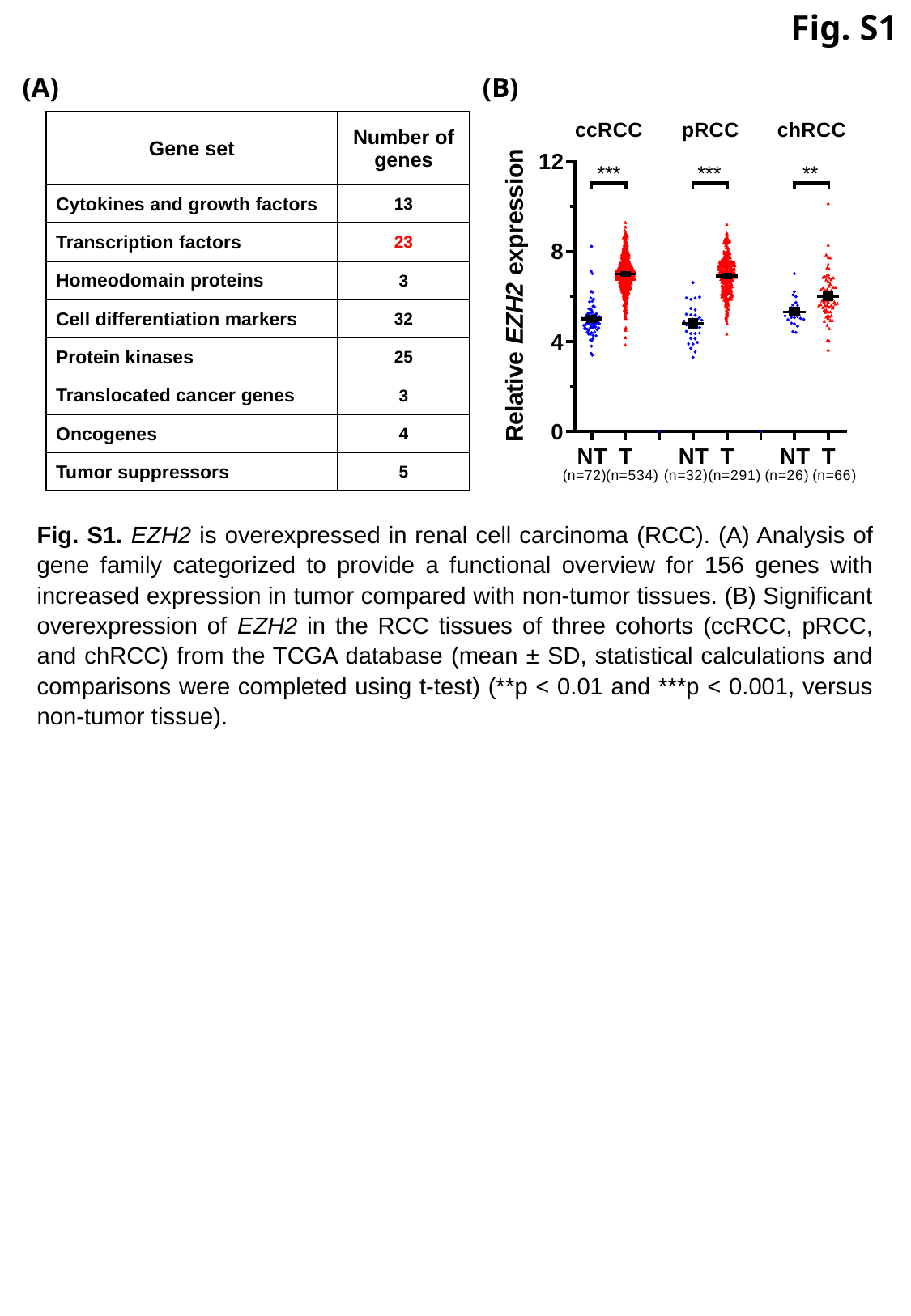

Fig. S1
(B)
(A)
| Gene set | Number of genes |
| --- | --- |
| Cytokines and growth factors | 13 |
| Transcription factors | 23 |
| Homeodomain proteins | 3 |
| Cell differentiation markers | 32 |
| Protein kinases | 25 |
| Translocated cancer genes | 3 |
| Oncogenes | 4 |
| Tumor suppressors | 5 |
Fig. S1. EZH2 is overexpressed in renal cell carcinoma (RCC). (A) Analysis of gene family categorized to provide a functional overview for 156 genes with increased expression in tumor compared with non-tumor tissues. (B) Significant overexpression of EZH2 in the RCC tissues of three cohorts (ccRCC, pRCC, and chRCC) from the TCGA database (mean ± SD, statistical calculations and comparisons were completed using t-test) (**p < 0.01 and ***p < 0.001, versus non-tumor tissue).

## Slide 2
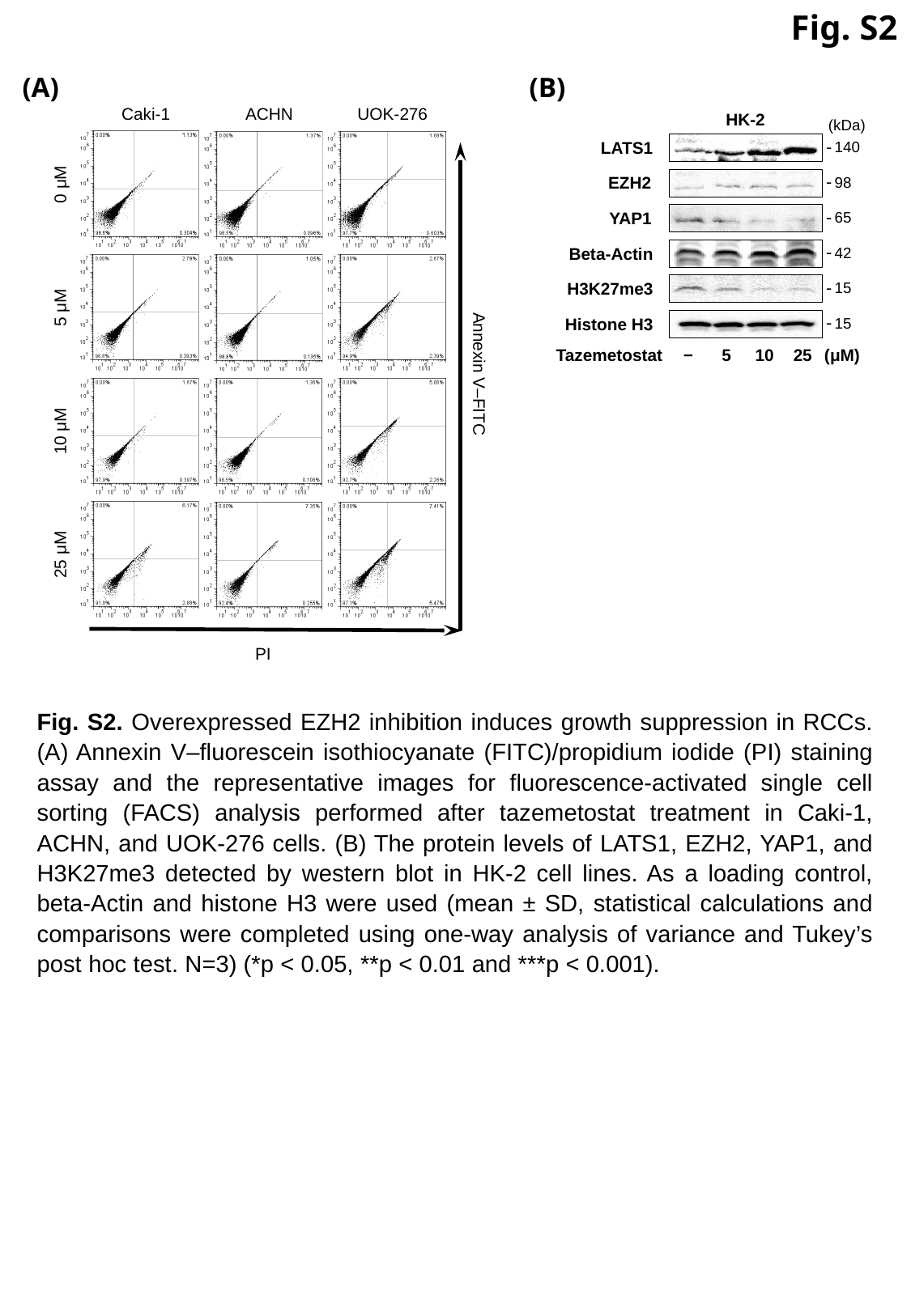

Fig. S2
(A)
(B)
Caki-1
ACHN
UOK-276
0 μM
5 μM
Annexin V–FITC
10 μM
25 μM
PI
HK-2
(kDa)
LATS1
-
140
EZH2
-
98
YAP1
-
65
Βeta-Actin
-
42
H3K27me3
-
15
Histone H3
-
15
Tazemetostat
−
5
10
25
(μM)
Fig. S2. Overexpressed EZH2 inhibition induces growth suppression in RCCs. (A) Annexin V–fluorescein isothiocyanate (FITC)/propidium iodide (PI) staining assay and the representative images for fluorescence-activated single cell sorting (FACS) analysis performed after tazemetostat treatment in Caki-1, ACHN, and UOK-276 cells. (B) The protein levels of LATS1, EZH2, YAP1, and H3K27me3 detected by western blot in HK-2 cell lines. As a loading control, beta-Actin and histone H3 were used (mean ± SD, statistical calculations and comparisons were completed using one-way analysis of variance and Tukey’s post hoc test. N=3) (*p < 0.05, **p < 0.01 and ***p < 0.001).

## Slide 3
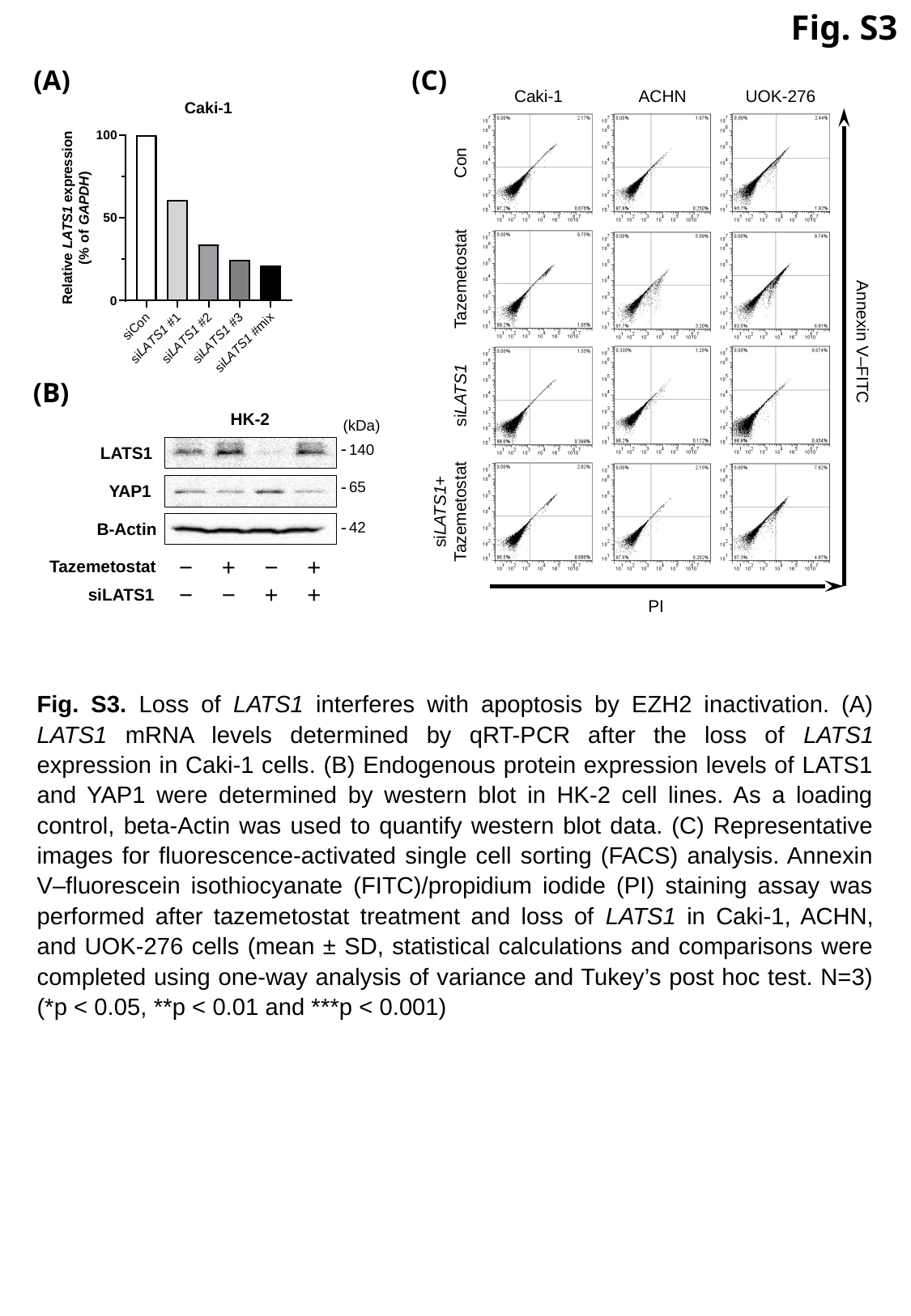

Fig. S3
(A)
(C)
Caki-1
ACHN
UOK-276
Con
Tazemetostat
Annexin V–FITC
siLATS1
siLATS1+
Tazemetostat
PI
(B)
HK-2
(kDa)
-
140
LATS1
-
65
YAP1
Β-Actin
-
42
−
+
−
+
Tazemetostat
−
−
+
+
siLATS1
Fig. S3. Loss of LATS1 interferes with apoptosis by EZH2 inactivation. (A) LATS1 mRNA levels determined by qRT-PCR after the loss of LATS1 expression in Caki-1 cells. (B) Endogenous protein expression levels of LATS1 and YAP1 were determined by western blot in HK-2 cell lines. As a loading control, beta-Actin was used to quantify western blot data. (C) Representative images for fluorescence-activated single cell sorting (FACS) analysis. Annexin V–fluorescein isothiocyanate (FITC)/propidium iodide (PI) staining assay was performed after tazemetostat treatment and loss of LATS1 in Caki-1, ACHN, and UOK-276 cells (mean ± SD, statistical calculations and comparisons were completed using one-way analysis of variance and Tukey’s post hoc test. N=3) (*p < 0.05, **p < 0.01 and ***p < 0.001)
